# Listeriolysin O Regulates the Expression of Optineurin, an Autophagy Adaptor That Inhibits the Growth of *Listeria monocytogenes*

**DOI:** 10.3390/toxins9090273

**Published:** 2017-09-05

**Authors:** Madhu Puri, Luigi La Pietra, Mobarak Abu Mraheil, Rudolf Lucas, Trinad Chakraborty, Helena Pillich

**Affiliations:** 1 Institute of Medical Microbiology, Justus-Liebig University, 35392 Giessen, Germany; madhu.006.2009@gmail.com (M.P.); luigi.la-pietra@mikrobio.med.uni-giessen.de (L.L.P.); mobarak.mraheil@mikrobio.med.uni-giessen.de (M.A.M.); 2 Vascular Biology Center, Department of Pharmacology and Toxicology and Division of Pulmonary and Critical Care Medicine, Medical College of Georgia, Augusta University, Augusta, GA 30912, USA; rlucas@augusta.edu

**Keywords:** listeriolysin O, *Listeria monocytogenes*, optineurin, autophagy

## Abstract

Autophagy, a well-established defense mechanism, enables the elimination of intracellular pathogens including *Listeria monocytogenes*. Host cell recognition results in ubiquitination of *L*. *monocytogenes* and interaction with autophagy adaptors p62/SQSTM1 and NDP52, which target bacteria to autophagosomes by binding to microtubule-associated protein 1 light chain 3 (LC3). Although studies have indicated that *L*. *monocytogenes* induces autophagy, the significance of this process in the infectious cycle and the mechanisms involved remain poorly understood. Here, we examined the role of the autophagy adaptor optineurin (OPTN), the phosphorylation of which by the TANK binding kinase 1 (TBK1) enhances its affinity for LC3 and promotes autophagosomal degradation, during *L*. *monocytogenes* infection. In LC3- and OPTN-depleted host cells, intracellular replicating *L*. *monocytogenes* increased, an effect not seen with a mutant lacking the pore-forming toxin listeriolysin O (LLO). LLO induced the production of OPTN. In host cells expressing an inactive TBK1, bacterial replication was also inhibited. Our studies have uncovered an OPTN-dependent pathway in which *L*. *monocytogenes* uses LLO to restrict bacterial growth. Hence, manipulation of autophagy by *L*. *monocytogenes*, either through induction or evasion, represents a key event in its intracellular life style and could lead to either cytosolic growth or persistence in intracellular vacuolar structures.

## 1. Introduction

*Listeria monocytogenes* is a Gram-positive, ubiquitously distributed, facultative intracellular pathogen that causes listeriosis, a lethal food-borne disease. Following invasion into host cells, the pathogen breaches single-membrane vacuolar compartments to escape into the cytosol using listeriolysin O (LLO) and/or its phospholipases [1,2]. Subsequently, cytosolic bacteria employ the surface protein actin-assembly inducing protein (ActA) to recruit components of the host-cell actin machinery to facilitate intracellular bacterial movement and cell-to-cell spread [1]. However, there is increasing evidence to suggest that a proportion of the bacteria modulate, via LLO, their vacuolar compartments to enable replication and propagation [3,4].

LLO is a cholesterol-dependent cytolysin (CDC) that inserts into host plasma membranes to form pores, thereby inducing host cell signaling cascades that regulate repair processes such as autophagy [5]. LLO is also required for the entry of *L*. *monocytogenes* into autophagosomal compartments, which fuse with lysosomes, eventually leading to enzymatic degradation [6,7].

Autophagy is a cellular degradation system that involves the enclosure of cargo molecules in double-membrane vacuoles called autophagosomes and their subsequent degradation by lysosomal hydrolases. Autophagic cargo can be comprised of damaged cellular organelles, protein aggregates or pathogens [8]. Autophagy can be triggered by amino acid starvation, low cellular energy levels, withdrawal of growth factors, hypoxia, oxidative stress, endoplasmic reticulum (ER) stress, damaged cellular organelles and infection. Autophagy is an essential part of cellular homeostasis, and an indispensable cellular defense mechanism against intracellular pathogens [8].

Three types of autophagy can occur in cells. Macro-autophagy is the entrapment of cytoplasmic cargo into autophagosomes, followed by fusion with lysosomes leading to subsequent cargo degradation. Micro-autophagy comprises the direct lysosomal uptake of cytosolic components by the invagination of the lysosomal membrane. Chaperone-mediated autophagy involves chaperone proteins that are recognized by the lysosomal membrane receptor lysosome-associated membrane protein 2A. These chaperone proteins form a complex with cargo and are translocated across the lysosomal membrane [9]. Autophagy can also be classified as selective and non-selective. Selective autophagy is mediated by autophagy adaptors or cargo receptors that specifically recognize cargo for degradation, whereas, in non-selective autophagy, cargo is indiscriminately cloistered into developing autophagosomes [10]. Xenophagy is a term used to describe the selective autophagy of intracellular pathogens. In this article, the term “autophagy” refers to the process of xenophagy.

Following induction of autophagy, the cytosol-bound form of microtubule-associated protein 1 light chain 3 (MAP1LC3 or LC3) is converted by the autophagy related proteins (ATG) into its lipidated membrane-bound form, i.e., from LC3-I to LC3-II, by phosphatidylethanolamine conjugation [11]. Autophagy is an innate immune system that restricts the replication of many intracellular pathogens, which include *Salmonella typhimurium*, *Mycobacterium tuberculosis*, *Streptococcus pyogenes* and *Streptococcus pneumoniae* [12,13,14,15]. This process requires autophagy adaptors that specifically and selectively recognize intracellular bacteria for their degradation [9]. Autophagy adaptors are characterized by the presence of a ubiquitin-binding domain (UBD) that recognizes ubiquitinated cargo, and an LC3-interacting region (LIR) that links this cargo to the autophagosomal membrane [16]. However, pathogens such as *S*. *typhimurium* and *L*. *monocytogenes* have evolved sophisticated strategies to either suppress autophagy, or even prevent recognition and subsequent capture by the autophagic machinery [17,18,19].

Previous studies have shown that two autophagy adaptors, sequestosome 1 (SQSTM1, also known as p62) and calcium binding and coiled-coil domain 2 (CALCOCO2, also known as NDP52), interact in an exchangeable manner with *L*. *monocytogenes* to target bacteria to autophagosomal compartments [17,18]. During an examination of the host transcriptional response to LLO, we noted clear and reproducible upregulation of optineurin (OPTN) at very early time points [20]. OPTN has been implicated in many signaling pathways and cellular processes, but, in recent years, its role in autophagy has attracted particular attention. OPTN is now recognized as a member of autophagy adaptors that link LC3 (through their LIR motif) to ubiquitinated cargo (via their UBD) [16]. In the case of *S*. *typhimurium*, OPTN has been shown to associate with ubiquitinated intracellular bacteria and recruit the TANK binding kinase 1 (TBK1), which enhances OPTN activity [21]. 

Here, we examined the role of OPTN and the potential interaction between LLO and OPTN in autophagy of *L*. *monocytogenes*. We show that LLO induces the upregulation of OPTN in HeLa cells. The activation of OPTN was required to restrict the growth of intracellular *L*. *monocytogenes* wild-type (wt). By contrast, OPTN played no role in the growth restriction of the LLO-negative mutant, the latter of which was able to escape from vacuoles and reach the cytoplasm. Our data demonstrate that OPTN targets *Listeria* to degradation in an LLO-dependent manner. 

## 2. Results

### 2.1. LC3 Is Essential for the Intracellular Growth Restriction of LLO-Producing *L. monocytogenes*

**Figure 1:**
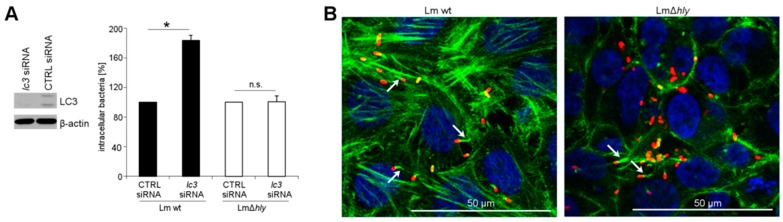
(**A**) Growth of *L*. *monocytogenes* wt and LLO-negative mutant (∆*hly*) in LC3-depleted HeLa cells. LC3 depletion was confirmed by immunoblotting of cell lysates, using β-actin as loading control; (**B**) localization of intracellular bacteria as determined by immunofluorescence microscopy. *Listeria* = red, actin cytoskeleton = green, nucleus = blue. Arrows indicate bacterial actin-tails. * *p* < 0.05 vs. CTRL; n.s.: not significant.

In HeLa cells, depletion of the autophagy factor LC3 resulted in a significant increase in intracellular replicating wt *L*. *monocytogenes* (Figure 1A). These cells are, however, also permissive for the replication of LLO-negative mutants, which exited into the cytoplasm and formed actin tails (Figure 1B). However, depletion of LC3 did not affect the intracellular numbers of LLO-negative *L*. *monocytogenes* (Figure 1A). 

### 2.2. LLO Upregulates OPTN in HeLa Cells

**Figure 2:**
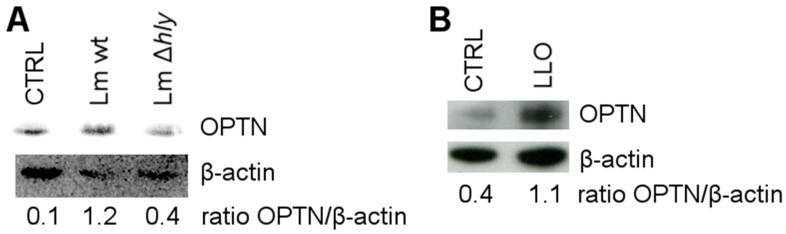
(**A**) Immunoblotting for OPTN in HeLa cells infected with *L*. *monocytogenes* wt and LLO-negative mutant (∆*hly*) or left uninfected (CTRL) for 6 h, with β-actin as loading control; (**B**) immunoblotting for OPTN in HeLa cells treated with LLO or left untreated (CTRL), with β-actin as loading control.

As LLO-producing *L*. *monocytogenes* were targeted by the autophagy factor LC3, we examined whether LLO regulates OPTN activity. To that purpose, HeLa cells were infected with *L*. *monocytogenes* wt and LLO-negative mutant. OPTN levels were determined by immunoblotting. As can be seen in Figure 2A, OPTN was upregulated in cells infected with *L*. *monocytogenes* wt but not *L*. *monocytogenes* ∆*hly*, indicating that LLO is required to regulate the expression of OPTN. To confirm this result, HeLa cells were treated with lipopolysaccharide (LPS)-free LLO, purified from *L. innocua*, and changes in OPTN expression were analyzed by immunoblotting. Indeed, LLO significantly induced the upregulation of OPTN (Figure 2B). 

### 2.3. OPTN Phosphorylation by TBK1 Is Essential for the Growth Restriction of *L. monocytogenes*

**Figure 3:**
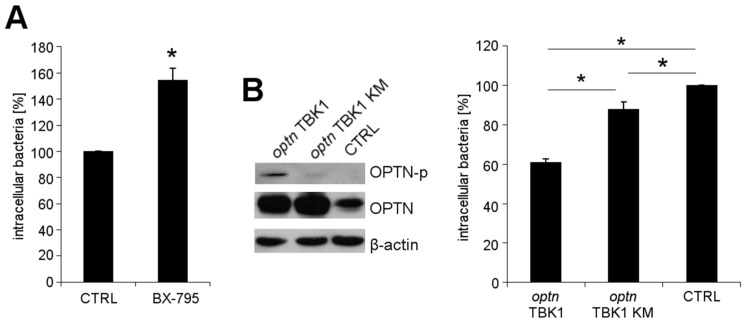
*L*. *monocytogenes* wt growth in HeLa cells (**A**) treated with BX-795 prior to infection and (**B**) transfected with a plasmid encoding (1) OPTN and TBK1 wt; (2) OPTN and TBK1 with an ineffective kinase (KM); and (3) an empty vector (CTRL). The phosphorylation of OPTN was confirmed by immunoblotting using antibodies against phosphorylated OPTN (OPTN-p), OPTN and β-actin (loading control). * *p* < 0.05 vs. CTRL.

Phosphorylation of OPTN by TBK1 enhances its affinity for LC3 [21]. To elaborate the role of TBK1 in *L*. *monocytogenes* growth restriction, TBK1 was inhibited with a reversible inhibitor BX-795, prior to infection of HeLa cells with wt *L*. *monocytogenes*. Increased intracellular numbers of wt *L*. *monocytogenes* were observed in cells treated with BX-795, as compared to untreated control cells (Figure 3A). The treatment of *L*. *monocytogenes* wt with BX-795 did not affect bacterial viability (Figure S1). 

Because TBK1 also phosphorylates other autophagy adaptors, besides OPTN [22], we examined the role of phosphorylated OPTN in *L*. *monocytogenes* wt growth restriction in greater detail. Cells were co-transfected with a plasmid encoding (1) OPTN and TBK1; or (2) OPTN with TBK1 with an inactive kinase (KM) domain; and (3) an empty vector vehicle. OPTN reduced intracellular wt *L*. *monocytogenes* growth in the presence of active TBK1, but this effect was absent with the inactive TBK1 variant (Figure 3B). Thus, these results indicate that active TBK1 and phosphorylated OPTN are required to restrict the intracellular growth of *L*. *monocytogenes*.

### 2.4. The Reduction of OPTN Promotes the Growth of Wt *L. monocytogenes* in an LLO-Dependent Manner

**Figure 4:**
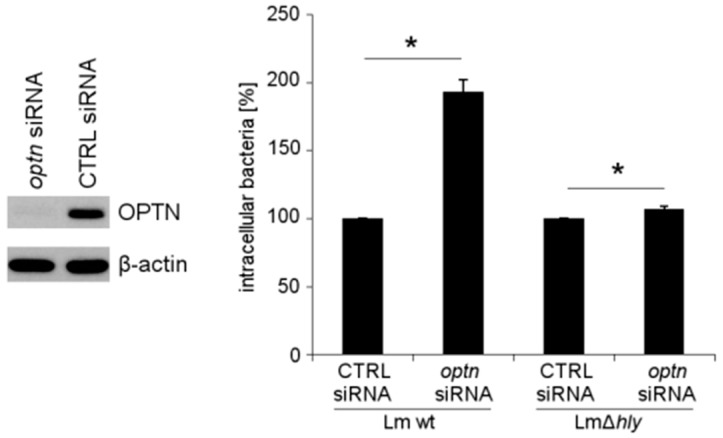
Growth of wt *L*. *monocytogenes* and LLO-negative mutant (∆*hly*) in OPTN-depleted HeLa cells. OPTN depletion was confirmed by immunoblotting of cell lysates with β-actin as loading control. * *p* < 0.05 vs. CTRL.

To determine the involvement of LLO in OPTN-mediated growth restriction of *L*. *monocytogenes*, we reduced expression of *optn* with specific siRNA in HeLa cells, and subsequently infected them with wt *L*. *monocytogenes* and its isogenic LLO-negative mutant ∆*hly*. In OPTN-depleted cells, the intracellular numbers of wt *L*. *monocytogenes* were significantly increased. By contrast, OPTN depletion did not affect the intracellular growth of *L*. *monocytogenes* ∆*hly* (Figure 4). This result implies that LLO production is essential for the growth restriction of *L*. *monocytogenes* by OPTN.

## 3. Discussion

Autophagy plays a crucial role in the clearance of intracellular *L*. *monocytogenes* [7,23]. Cytosolic *Listeria* are ubiquitinated and are subsequently detected by the autophagy adaptors SQSTM1 and NDP52, which target them to autophagosomes for degradation [17,18]. Current studies have focused on the question of how *L*. *monocytogenes* evades autophagic recognition and have provided insight that these bacteria use mimicry, i.e., coating themselves with components of the host cell cytoskeleton by means of ActA [17,24,25,26]. Our results in this study reveal another aspect of autophagic recognition. Indeed, we report that the autophagy adaptor OPTN is upregulated in response to LLO treatment. Significantly, OPTN reduces the intracellular growth of wt *L*. *monocytogenes*, but not that of its isogenic LLO-negative mutant strain. Detailed analysis has indicated that TBK1-mediated phosphorylation of OPTN is a crucial event in the restriction of intracellular growth of wt *L*. *monocytogenes*.

Previous studies on autophagosomal degradation of *L*. *monocytogenes* have shown that cytoplasmic bacteria are targeted by the autophagosomal machinery [23]. Other reports have demonstrated that LLO is required for autophagy induction, and it was postulated that *L*. *monocytogenes* containing phagosomes damaged by LLO might be targeted by autophagy [6,7]. The data reported in this study, for the first time, provide evidence that LLO induces the upregulation of the autophagy adaptor OPTN. We used HeLa cells to determine the role of OPTN during *L*. *monocytogenes* infection. This cell line is particularly well-suited for this study, since expression of LLO is dispensable for bacterial vacuolar escape in these cells [2], as evidenced by the presence of cytoplasmic LLO-negative *L*. *monocytogenes* with actin tails.

**Figure 5:**
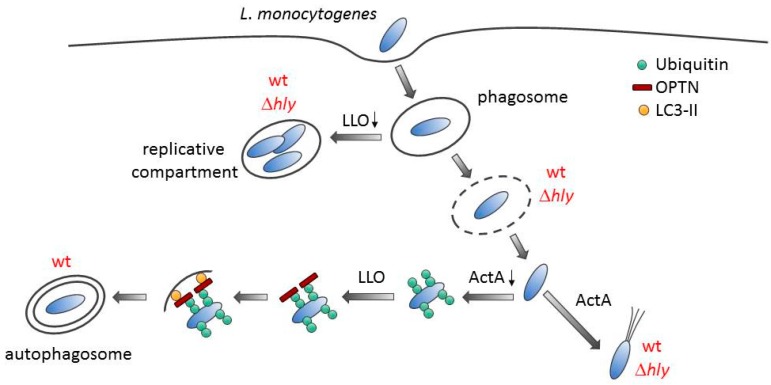
A model for autophagy induction during *L*. *monocytogenes* infection. After entry, *L*. *monocytogenes* is trapped within a single-membrane vacuole. Listeriolysin O (LLO)-negative mutant (∆*hly*) or wild type (wt) bacteria expressing low levels of LLO allow the establishment of a replicative niche, which cannot be autophagocytosed. However, the pathogen escapes from the vacuolar compartment with the help of phospholipases into the cytosol. Cytoplasmic bacteria expressing ActA recruit the host actin cytoskeleton machinery and are camouflaged from autophagic recognition. In contrast, bacteria that do not quickly express ActA are ubiquitinated. This is followed by the binding of ubiquitinated bacteria to OPTN, whose expression is induced by LLO. OPTN interacts with LC3-containing membranes, leading to autophagosome formation around the bacterium.

Our data show that intracellular growing *L*. *monocytogenes* consist of two populations: one which generates LLO and may be targeted by autophagy, thereby leading to its intracellular growth restriction, and a second group that might not be targeted for autophagic clearance and therefore, its growth remains unrestricted. These data therefore suggest that, in addition to evasion of autophagy by ActA [17], *L*. *monocytogenes* may also manipulate the cellular autophagic machinery by induction through LLO, to promote its growth and persistence in host cells. Thus, bacteria that escape the vacuole and hyper-replicate in the host cytosol may be subjected to autophagic detection and removal (Figure 5). Further studies are required to conclude that autophagy is involved in the growth restriction of LLO-producing *L. monocytogenes* under these experimental conditions. It appears counterintuitive that LLO induces the upregulation of the autophagy adaptor protein OPTN. However, other functions of OPTN may be of relevance here, as it has been shown that the OPTN-TBK1 complex leads to the phosphorylation, dimerization, and nuclear localization of the interferon regulatory factor 3 (IRF3), which, in turn, mediates the transcription of the interferon (IFN) type 1 response genes [27]. Secreted IFNα/β would stimulate the production of more potent antimicrobial interferon IFNγ by bystander cells, subsequently leading to cell-autonomous bacterial killing [28,29]. Thus, our results suggest that LLO induces a host response, the upregulation of OPTN, which is required to detect and to degrade intracellular *L*. *monocytogenes*. 

To date, only one additional bacterial pathogen, namely *S*. *typhimurium,* was shown to be targeted by OPTN for its autophagosomal degradation [21]. For *Salmonella*, it was demonstrated that LPS leads to TBK1-dependent phosphorylation of OPTN [21], which is a function shared with the proteinaceous toxin LLO. During *S*. *typhimurium* infection, these bacteria remodel the phagosome into a non-degradative compartment referred to as *Salmonella*-containing vacuole (SCV) [30]. In autophagy-deficient cells, infection with *S*. *typhimurium* leads to a loss of membrane integrity in SCVs, thus suggesting that autophagy may be involved in membrane repair [31]. There is currently little evidence for repair of host membranes by the autophagic machinery and this certainly requires further investigation. 

Our data presented here imply that a quantitative assessment of bacterial replication does not distinguish between the different compartments occupied by the bacterium during intracellular growth. Thus, the compartment in which LLO-deficient bacteria grow in infected cells is not targeted for autophagy and may indeed be the spacious *Listeria*-associated phagosomes previously described, where *L*. *monocytogenes* grow, albeit at low replication rates [3]. Further studies are warranted to examine replicative niches of *L*. *monocytogenes* and their contribution to overall growth.

## 4. Conclusions

In conclusion, host cells employ OPTN to control the intracellular growth of *L. monocytogenes* via host signaling that is activated by LLO. LLO belongs to the family of CDCs, which are mainly produced by Gram-positive bacteria including species from the genera *Arcanobacterium*, *Bacillus*, *Clostridium*, *Gardnerella*, *Lactobacillus*, *Listeria* and *Streptococcus* [32]. Recently, it was demonstrated that *S*. *pneumoniae* induces autophagy in a pneumolysin (a CDC)-dependent manner [15]. It might be worth analyzing as to whether or not this toxin activates autophagy via OPTN, as well, which would suggest a general mechanism of CDC-dependent autophagic induction. 

## 5. Materials and Methods

### 5.1. Cell Culture

HeLa (human cervical adenocarcinoma) cells were cultured in Dulbecco’s modified Eagle medium (DMEM) (Thermo Fischer Scientific, Waltham, MA, USA) supplemented with 10% fetal bovine serum (FBS) (Biochrom, Berlin, Germany) at 37 °C in a humidified, 5% CO_2_-air atmosphere. The cells were seeded in cell culture dishes with medium containing 10% FBS 24 h prior to the experiments. At 90–100% confluency, the cells were washed once with Hanks’ Balanced Salt Solution (HBSS) (Biochrom, Berlin, Germany), and incubated in DMEM containing 10% FBS for 2 h. The cells were then again washed three times with HBSS, and infected in medium containing 0.5% FBS. The cells were incubated in medium containing 0.5% FBS throughout the duration of infection. For treatment with 50 ng/mL LLO, the cells were washed five times with HBSS and incubation with LLO was performed in medium without FBS for 1 h. Prior to treatment, LLO was activated by incubation with 5 mM dithiothreitol (Sigma-Aldrich, St. Louis, MO, USA) for 10 min at room temperature (RT). LLO was isolated and purified from *Listeria innocua* expressing LLO as described [33]. 

The treatment of cells with 1 μM BX-795 (Merck Millipore, Billerica, MA, USA) was performed 1 h before infection in medium containing 0.5% FBS. The infection was done in the medium containing BX-795.

### 5.2. RNAi Transfection

The cells were plated shortly before transfection in 1.1 mL DMEM containing 10% FBS. The siRNA (5 nM for *lc3*; 10 nM for *optn*) and the HiPerFect reagent (1.5 μL for *lc3*; 3 μL for *optn*) were diluted in 100 μL DMEM and incubated for 5 min at RT. The transfection complexes were added dropwise to the cells, and the cells were incubated for 48 h. Subsequently, the cells were washed three times with HBSS to terminate the transfection, and DMEM containing 10% FBS was added. The cells were then infected as described. *lc3* (SI02655597), *optn* (SI00132020) and scrambled (1022076) siRNA were purchased from Qiagen (Hilden, Germany).

### 5.3. Plasmid Transfection

HeLa cells were plated in 24-well plates one day before transfection. Shortly before transfection, the cells were washed five times with sterilized HBSS, incubated in medium without FBS and subsequently transfected with the plasmid pcDNA3.1(+)/HA-OPTN, pcDNA3.1-TBK1-myc-His6, pcDNA3.1-TBK1-myc-His6 KM [21] and the empty vector pRK5 as control (BD Biosciences, Franklin Lakes, NJ, USA). The plasmid DNA (0.95 μg/well) and Lipofectamine 2000 (Invitrogen, Carlsbad, CA, USA; 3 μL/well) were diluted in Opti-MEM I (Thermo Fischer Scientific, Waltham, MA, USA), and equal volumes of both were combined and incubated for 20 min at RT. The plasmid DNA-Lipofectamine 2000 complexes were added to the cells (100 μL/well), and incubated at 37 °C for 4 h. Later, fresh DMEM containing 10% FBS was added, and the cells were infected after 24 h. 

### 5.4. Bacterial Culture and Infection

*L*. *monocytogenes* wt (EGD-e) [34] and *L*. *monocytogenes* Δ*hly* (a mutant lacking LLO) [35] were grown in Brain–Heart–Infusion (BHI) medium. *Escherichia coli* Top 10 (Invitrogen) were cultured in Luria–Bertani medium. The bacteria were grown with constant shaking (180 rpm) at 37 °C. For infection, overnight grown cultures of *L*. *monocytogenes* were diluted (1:50) in BHI medium, and cultured to exponential growth phase as determined by the optical density at 600 nm. An appropriate culture volume was centrifuged at 13,000 rpm for 1 min at RT. The bacterial pellet was washed twice with HBSS, resuspended in DMEM containing 0.5% FBS, and used for infection. A multiplicity-of-infection of 10 was used for infection. For determination of intracellular bacterial number, the extracellular bacteria were eliminated 1 h post infection (p.i.) by the incubation of the infected cells in DMEM containing 10% FBS, and 50 μg/mL of gentamicin. For analysis of OPTN levels, cells were infected for 6 h without gentamicin treatment.

### 5.5. Determination of the Number of Intracellular Bacteria

Four hours p.i., the cells were washed three times with phosphate-buffered saline (PBS; pH 7.4), and lysed with cold water containing 0.2% Triton X-100 for 20 min at RT. The bacteria were diluted in PBS and plated on BHI agar plates. 

### 5.6. Protein Preparation from Eukaryotic Cells and Immunoblotting

Cell lysis was performed with RIPA [33] or CHAPS lysis buffer purchased from ProteinSimple (San Jose, CA, USA) [36]. The total protein content was measured with bicinchoninic acid solution (Sigma-Aldrich, St. Louis, MO, USA) assay.

Equal amounts of proteins were analysed by immunoblotting [33]. Antibodies against β-actin (#4970, Cell Signaling Technology, Danvers, MA, USA), LC3 (#sc-16755, Santa Cruz Biotechnology, Dallas, TX, USA), OPTN (#10837-1-AP, Proteintech, Chicago, IL, USA) and phosphorylated-OPTN [21] were used. HRP-conjugated goat anti-rabbit IgG (#sc-2004) and donkey anti-goat IgG (#sc-2020) were purchased from Santa Cruz Biotechnology. 

### 5.7. Immunofluorescence

The cells cultured on coverslips were infected. Four hours p.i., the cells were washed three times with PBS, fixed in 3.7% formaldehyde-PBS for 20 min at RT and incubated with immunofluorescence buffer (0.3% Triton-X-100, 1% BSA in PBS) at RT. After incubation with monoclonal primary anti-*Listeria* antibody (M108, undiluted) overnight at 4 °C, the cells were washed three times with PBS and incubated with 1:1000 anti-mouse IgG Fab2 Fragment Alexa Fluor 647-conjugated secondary antibody (Cell Signaling Technology, Danvers, MA, USA, #4410) and 1:40 Alexa Fluor 488-conjugated phalloidin (Thermo Fisher Scientific, Waltham, MA, USA, #A12379) for 2 h at 37 °C in the dark. After three washing steps, the coverslips were mounted with ProLong Gold antifade reagent with DAPI (Thermo Fisher Scientific, Waltham, MA, USA, #P36935) and imaged by confocal microscopy (Leica TCS SP5, Leica Microsystems, Wetzlar, Germany).

### 5.8. Statistical Analysis

Statistical analysis of experiments was performed with SigmaPlot 11 (Systat Software, San Jose, CA, USA). The data of Figure 1A, Figure 3A, Figure 4 and Figure S1 were analyzed by *t*-test. The data of Figure 3B were analyzed by one-way ANOVA with Tukey. Mean values ± SEM are plotted from three independent experiments. Representative immunofluorescence or immunoblotting images from three independent experiments are shown.

## Supplementary Materials

The following are available online at www.mdpi.com/2072-6651/9/9/273/s1, Figure S1: BX-795 has no effect on bacterial viability. *L*. *monocytogenes* wt was added to the cell culture media (without cells) containing 1 μM BX-795. The bacteria were plated after 1 h.
