# Supplementary material for: Listeriolysin O Regulates the Expression of Optineurin, an Autophagy Adaptor That Inhibits the Growth of Listeria monocytogenes
Source: Toxins (Basel). 2017 Sep 5;9(9):273. doi: 10.3390/toxins9090273 (PMC5618206; doi:10.3390/toxins9090273)
Supplement: Supplementary file 1 [file toxins-09-00273-s001.pdf]

Listeriolysin O Regulates the Expression of Optineurin, an Autophagy Adaptor that Inhibits the Growth of *Listeria monocytogenes*

Madhu Puri, Luigi La Pietra, Mobarak Abu Mraheil, Rudolf Lucas, Trinad Chakraborty and Helena Pillich

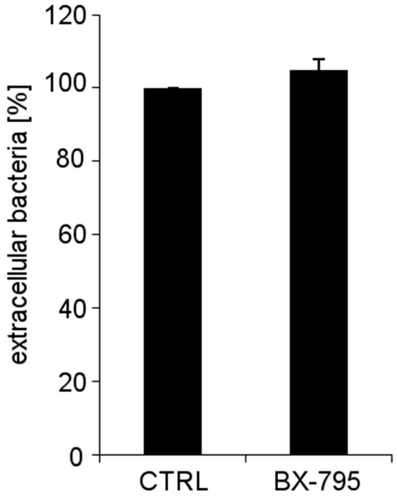

Figure S1. BX-795 has no effect on bacterial viability. *L. monocytogenes* wt was added to the cell culture media (without cells) containing 1 μM BX-795. The bacteria were plated after 1 h.
